# Supplementary material for: Endogenous Levels of Gamma Amino-Butyric Acid Are Correlated to Glutamic-Acid Decarboxylase Antibody Levels in Type 1 Diabetes
Source: Biomedicines. 2021 Dec 31;10(1):91. doi: 10.3390/biomedicines10010091 (PMC8773285; doi:10.3390/biomedicines10010091)
Supplement: Supplementary file 1 [file biomedicines-10-00091-s001.zip › biomedicines-1510246-supplementary.pdf]

Supplementary Table S1 – Analyzed circulating cytokines

**Catalog #HCYP4MAG-64K**	**HC** (n=27)	**T1D** (n=56)
Interferon-beta (pg/mL)	705 ± 105	608 ± 79
IL-19 (pg/mL)	708 ± 32	709 ± 14
IL-24 (ng/mL)	n/a	n/a
IL-34 (pg/mL)	n/a	n/a
IL-35 (ng/mL)	n/a	n/a
IL-36 beta (pg/mL)	6.2 ± 0.8	6.5 ± 0.6
IL-37 (pg/mL)	21.3 ± 4.3	19.2 ± 3.0
IL-38 (pg/mL)	55.9 ±6.6	52.1 ± 6.0
**Catalog #HTH17MAG-14K**		
TNF-alpha (pg/mL)	57.6 ± 4.5	64.3 ± 3.6
TNF-beta	0.20 ± 0.06	0.23 ± 0.06
Interferon-gamma (pg/mL)	181 ± 24	176 ± 9
IL-1 beta (pg/mL)	17.1 ± 5.9	13.0 ± 0.98
IL-2 (pg/mL)	41.9 ± 16.4	31.4 ± 2.1
IL-4 (ng/mL)	0.5 ± 0.07	0.5 ±0.05
IL-5 (pg/mL)	56.3 ± 7.8	58.5 ± 5.7
IL-6 (pg/mL)	49.2 ± 12.7	54.2 ± 12.6
IL-9 (pg/mL)	61.9 ± 11.0	60.0 ± 4.8
IL-10 (pg/mL)	29.2 ± 3.4	31.9 ±2.7
IL-12p70 (pg/mL)	40.2 ± 7.1	38.1 ± 2.8
IL-13 (pg/mL)	142 ± 34	154 ± 31
IL-15 (pg/mL)	44.8 ± 7.0	45.5 ± 3.6
IL-17A (pg/mL)	42.6 ± 18.8	24.0 ± 2.0
IL-17E (ng/mL)	0.35 ± 0.19	0.20 ± 0.02
IL-17F (ng/mL)	0.075 ± 0.02	0.079 ± 0.009
IL-21 (pg/mL)	69.1 ± 10.1	67.7 ± 4.9
IL-22 (ng/mL)	n/a	n/a
IL-23 (ng/mL)	6.2 ± 1.1	7.7 ± 0.92
IL-27 (ng/mL)	1.3 ± 0.08	1.4 ± 0.07
IL-28A (ng/mL)	2.2 ± 0.36	2.2 ± 0.24
IL-31 (ng/mL)	0.13 ± 0.04	0.12 ± 0.01
IL-33 (pg/mL)	140 ± 14	161 ± 11

Circulating cytokines in plasma were analyzed with magnetic bead-based Luminex using two commercially available assays Merck Millipore (Burlington, MA, USA) according to the manufacturer's protocol. IL-22, IL-24, IL-34, and IL-35 were excluded from data analysis since >50% of the samples were below the detection level. Comparisons between the two

groups were performed using an unpaired two-tailed t-test. Data are presented as means ± SEM.
